# Validation of QTLs for Fiber Quality Introgressed from *Gossypium mustelinum* by Selective Genotyping

**DOI:** 10.1534/g3.120.401125

**Published:** 2020-05-11

**Authors:** Qi Chen, Wei Wang, Caixiang Wang, Mi Zhang, Jiwen Yu, Yifei Zhang, Baotong Yuan, Yunyun Ding, Don C. Jones, Andrew H. Paterson, Peng W. Chee, Baohua Wang

**Affiliations:** * School of Life Sciences, Nantong University, Nantong, Jiangsu 226019, P.R. China,; ^†^ Agricultural Science Institute of Coastal Region of Jiangsu, Yancheng, Jiangsu 224002, P.R. China,; ^‡^ State Key Laboratory of Cotton Biology, Anyang, Henan 455000, P.R. China,; ^§^ Cotton Incorporated, 6399 Weston Parkway, Cary, NC 27513,; ^**^ Plant Genome Mapping Laboratory, University of Georgia, Athens, GA 30602, and; ^††^ Department of Crop and Soil Sciences, University of Georgia, Tifton, GA 31793

**Keywords:** *Gossypium mustelinum*, introgression lines, fiber quality traits, QTL

## Abstract

Gene introgression from wild species has been shown to be a feasible approach for fiber quality improvement in Upland cotton. Previously, we developed an interspecific *G. mustelinum* × *G. hirsutum* advanced-backcross population and mapped over one hundred QTL for fiber quality traits. In the current study, a trait-based selective genotyping approach was utilized to prioritize a small subset of introgression lines with high phenotypic values for different fiber quality traits, to simultaneously validate multiple fiber quality QTL in a single experiment. A total of 75 QTL were detected by CIM and/or single-marker analysis, including 11 significant marker-trait associations (*P* < 0.001) and three putative associations (*P* < 0.005) also reported in earlier studies. The QTL that have been validated include three each for fiber length, micronaire, and elongation, and one each for fiber strength and uniformity. Collectively, about 10% of the QTL previously reported have been validated here, indicating that selective genotyping has the potential to validate multiple marker-trait associations for different traits, especially those with a moderate to large-effect detected simultaneously in one experimental population. The *G. mustelinum* alleles contributed to improved fiber quality for all validated loci. The results from this study will lay the foundation for further fine mapping, marker-assisted selection and map-based gene cloning.

Cotton (*Gossypium* L.) is the most important fiber crop in the world, supplying a majority of the natural fiber used in the textile industry. While four cotton species have been domesticated, the production and supply of cotton fiber is mostly from Upland cotton (*Gossypium hirsutum* L.). The innovation of modern textile technology and the general increased demand for better quality textile products by consumers are imposing higher requirements on cotton fiber quality (Zhu and Weir 1998). In general, the quality of cotton fiber is determined by the physical properties of individual fibers such as length, strength, and fineness (Zumba and Rodgers 2016). Fiber length is considered to be the most important property of cotton in raw material marketing and yarn processing (Kuang and Yu 2015). However, fiber strength and ‘micronaire’ (fineness) are increasingly being emphasized in determining the value of a cotton bale in the international market. Fiber strength determines the tenacity of the fiber, whereas micronaire values reflect both developmental maturity of cell walls and fiber fineness measured in linear density (Zumba and Rodgers 2016).

The coordinated improvement of fiber length, strength, and micronaire has become a primary objective of many cotton breeding programs. For example, China is one of the largest cotton producers in the world, with much raw cotton being consumed domestically by its textile industry to meet demand from customers locally and abroad. In recent years, cotton produced in China has experienced price disadvantage due to low fiber strength, high micronaire and poor combinations of fiber length and strength. Growers could benefit from new varieties having not only improved yield but also better fiber quality.

Although the domestication and modern breeding of Upland cotton has led to increased productivity and improved fiber quality, it has been accompanied by an extreme reduction in genetic diversity. Indeed, modern Upland cotton germplasm has very little genetic diversity with the current elite cultivars possessing a narrow range of fiber quality traits. Utilizing gene introgression from related species such as *Gossypium barbadense*, *G. tomentosum* and *G. darwinii*, and *G. mustelinum* has been shown to be a viable approach to expand genetic diversity by introducing novel alleles for fiber quality improvement (Paterson *et al.* 2003; Chee *et al.* 2005a,b; Jenkins *et al.* 2012; Wang *et al.* 2012a; Cao *et al.* 2014; Chen *et al.* 2018; Keerio *et al.* 2018; Brown *et al.* 2019; Li *et al.* 2019).

To ameliorate issues such as hybrid breakdown, linkage drag, and other undesirable consequences that are often encountered in segregating populations derived from interspecific crosses, several researchers have proposed backcrossing early generation hybrids to the adapted parent to generate introgression lines (ILs) each carrying only one to a few introgressed chromosome segments from the exotic parent (Eshed and Zamir 1994; Tanksley and Nelson 1996). Such IL populations have a number of advantages compared to recombinant inbred populations. For example, ILs can facilitate fine mapping of QTL, since the location of a QTL can be narrowed to a smaller genomic interval by evaluating a series of ILs that differ for overlapping regions of the genome (Paterson *et al.* 1990). In addition, since the amount of exotic chromatin retained in each IL is small, the phenotypic variation segregating in the IL population is reduced to a manageable level, and the ability to discern QTL with small phenotypic effects is increased. Finally, favorable exotic alleles identified in a specific IL can be easily transferred into elite varieties since they contain a low percentage of exotic chromatin, so ILs provide germplasm potentially useful in breeding programs (von Korff *et al.* 2004).

Indigenous to South America, *G. mustelinum* is genetically most distant from *G. hirsutum* in the allotetraploid clade, and therefore potentially a rich source of genetic novelty. Identifying key quantitative trait loci (QTL) for fiber quality and introducing an appropriate subset of favorable alleles from *G. mustelinum* could make a significant contribution to the long-term improvement of Upland cotton germplasm. Previously, we developed an interspecific population consisting of 3203 BC_3_F_2_ derived plants from 21 independently derived advanced-backcross *G. mustelinum* by *G. hirsutum* families (Wang *et al.* 2016a), seeking to introgress favorable alleles for fiber quality traits from *G. mustelinum* into Upland cotton (Wang *et al.* 2016a,b; Wang *et al.* 2017a,b). We investigated the transmission genetics of six fiber quality traits including fiber elongation, fiber uniformity index, fiber length, short fiber content, fiber strength and micronaire in BC_3_F_2_, BC_3_F_2:3_, and BC_3_F_2:4_ generations. For each trait, the mean values of some families outperformed the recurrent Upland parent, PD94042. A total of 131 QTL were detected for fiber elongation (24), fiber length (19), uniformity index (20), short fiber content (26), fiber strength (15) and micronaire (27). These results suggested that ILs carrying fiber quality alleles with positive effects from *G. mustelinum* could be extracted from families with improved fiber traits and utilized to improve Upland cotton.

Fiber quality QTL introgressed into Upland cotton from other species such as *G. barbadense* often have unexpected interactions with genetic backgrounds, even including opposite effects on different backgrounds (Chee *et al.* 2005a, 2005b; Draye *et al.* 2005) and environment-specific expression (Brown *et al.* 2019). Therefore, fiber quality QTL introgressed from exotic sources should be validated and evaluated in target environments before being deployed in marker-assisted breeding. In the current research, our objective was to validate the effects of fiber quality QTL previously identified in the advanced-backcross *G. mustelinum* × *G. hirsutum* population. We employed a trait-based selective genotyping approach, whereby analysis is conducted on a small subset of ILs with high phenotypic values targeting different fiber quality traits, to simultaneously validate multiple fiber quality QTL in a single experiment. The results from this research will lay the foundation for further fine mapping, marker-assisted selection and map-based gene cloning.

## Materials And Methods

### Population development and field evaluation

Previously, an interspecific advanced-backcross population was developed by crossing *G. mustelinum* acc. AD 4-8 with elite Upland germplasm line “PD94042” (PI 603219) released in 1998 by the USDA-ARS PeeDee breeding program in Florence, South Carolina. The parents of PD94042 include Jimian 8, developed by the Cotton Research Institute, Chinese Academy of Agricultural Sciences (May 1999). The F_1_ was backcrossed three times to PD94042 and 21 BC_3_F_1_ plants were self-pollinated to produce 21 independently derived BC_3_F_2_ families, consisting of 127-160 plants per family (3,203 plants in total). The DNA of BC_3_F_1_ plants was genotyped with 218 SSR markers approximately evenly distributed over the 26 chromosomes of the *G. hirsutum* ×*G. mustelinum* map (Wang *et al.* 2016a). DNA markers showing introgression from *G. mustelinum* in the BC_3_F_1_ were used to screen the entire BC_3_F_2_ family and utilized to map QTL for fiber elongation (Wang *et al.* 2016b). A subset of 12 families with size ranging from 130 to 160 (totally 1,826) lines was later advanced to BC_3_F_2:3_ and BC_3_F_2:4_ generations and utilized to map QTL for fiber length, strength, and fineness (Wang *et al.* 2017a,b).

In the current study, a selective genotyping population including 65 BC_3_F_2:4_ lines were selected based on two criteria to further validate QTL effects and positions. First, the *G. mustelinum* introgression segments from the 65 ILs provide broad coverage of the cotton genome (based on the BC_3_F_2_ genotypic data) and second, the ILs showed significant improvement for at least one fiber quality trait compared to the recurrent parent. A single seed from each of the selected ILs in the BC_3_F_2:4_ generation was planted in the greenhouse and advanced to BC_3_F_4:5_ generation. Seeds harvested from each BC_3_F_4:5_ plant were bulked and planted in peat pellets for germination in the greenhouse together with ten plots of the recurrent parent. Seedlings were hand-transplanted to a field in Yancheng, Jiangsu, China in April of 2011. The field experiment was a completely randomized design with two replications, and each plot was planted at approximately 30 cm between plants and 80 cm between the five-meter rows. At maturity, seed cotton was hand-harvested from all plots and ginned on a saw gin. Fiber length, strength, micronaire, uniformity index, and elongation were measured by an HVI900 fiber quality tester at Fiber Quality Supervising and Testing Center, Ministry of Agricultural and Rural Affairs, China. The field experiment was repeated in 2012 and 2013, thus providing a total of three environments.

### Genotyping and data analysis

In our prior QTL mapping study (Wang *et al.* 2016a,b; 2017a,b), genotyping was conducted on individual BC_3_F_2_ plants. Therefore, only 50% of the segregating loci were homozygous while the remaining 50% continued to segregate. To provide a more accurate genetic composition of each IL, a total of 1,629 pairs of SSR primers, including 391 selected from our previous research (Wang *et al.* 2016a,b), 238 EST-SSR primers from the NTU series developed by our laboratory (Wang *et al.* 2014, 2015), and 1,000 CICR primers developed by Institute of Cotton Research Institute of CAAS, were chosen to screen for polymorphisms between PD94042 and *G. mustelinum*. Polymorphic markers between the two parents were used to scan the IL population. A CTAB method was adopted for DNA extraction. A genetic linkage map was constructed with MapMaker 3.0 software (Wang *et al.* 2012b). Graphical genotypes representing the introgression of *G. mustelinum* in each IL were monitored by GGT2.0 (van Berloo 2008).

Mean values of fiber quality traits collected in two replicates were calculated for the ILs under each individual environment for QTL mapping. ANOVA was performed for the IL population under each environment, and significant differences existed among lines for all the traits. QTL analysis and estimation of various genetic parameters were conducted using composite interval mapping (CIM) implemented in the software WinQTLCart 2.5 (Wang *et al.* 2006). Mapping walk speed was set to 1 cM, and the LOD value of 3.0 was used as threshold for declaring the presence of QTL. In addition, associations with a LOD value of 2.0 and higher were reported as a putative QTL if detected in two environments simultaneously. Associations with fiber quality traits and each DNA marker individually (single-marker analysis) were tested for statistical significance by one-way ANOVA using the GLM procedure of SAS version 9.2 software package (SAS Institute 2008), and a significance threshold of *P* < 0.001.

### Data availability

The sequences of microsatellite markers for this project are available at CottonGen (https://www.cottongen.org/). Biometrical parameters of QTL affecting fiber quality traits by single-marker analysis are available in Table S1. The genotype data and phenotype data used to map QTL are available in File S1. Supplemental material available at figshare: https://doi.org/10.25387/g3.11799171.

## Results

### Phenotypic distribution of the ILs

**Figure 1:**
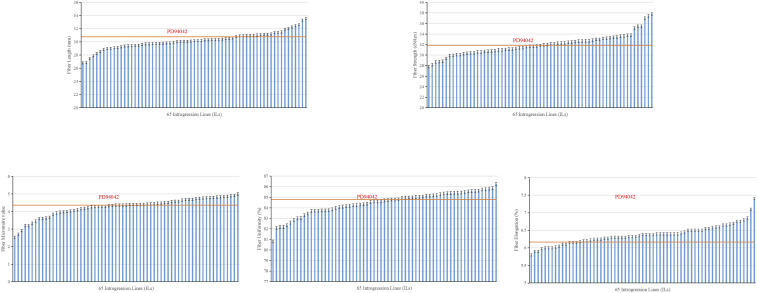
Phenotypic distribution of fiber quality traits in the ILs.

The distribution and descriptive statistics of the fiber quality traits are shown in Table 1 and the mean for each IL is presented in Figure 1. The fiber length of the ILs varied between 26.82 and 33.52 mm (Table 1), with an average of 30.21 mm and 13 ILs being superior to the recurrent Upland parent PD94042 (Figure 1a). The STR of the ILs ranged from 27.80 to 37.82 cN/tex (Table 1), with an average of 31.96 cN/tex and 31 ILs being superior to the recurrent parent (Figure 1b). The range of micronaire was between 2.54 and 5.00, and 17 ILs had lower MIC than the recurrent parent (Table 1). Generally micronaire readings of 3.7-4.2 are premium (A-level), 3.5-3.6 or 4.3-4.9 are base (B-level), and 3.4-and-under or 5.0-and-higher are substandard. Therefore, most ILs (56) had micronaire values at the A and B levels (3.5-4.9) (Figure 1c). The uniformity index of the ILs was from 80.80–86.25% (Table 1), with a mean value of 84.45% and 17 greater than the recurrent parent, most at approximately 85–86% (Figure 1d). The range of fiber elongation of ILs was 5.80-7.40, with a mean value of 6.37, and 51 higher than that of the recurrent parent (Figure 1e).

**Table 1:** Fiber quality components of *G. mustelinum* ILs

Trait*^a^*	Range	PD94042	Mean± SE.	CV (%)	Skewness
UHM (mm)	26.82-33.52	31.12	30.21 ± 0.16	0.04	−0.09
STR(cN/tex)	27.80-37.82	31.98	31.96 ± 0.25	0.06	0.68
MIC	2.54-5.00	4.61	4.26 ± 0.07	0.13	−1.28
UI (%)	80.80-86.25	85.24	84.45 ± 0.14	0.01	−1.00
ELO (%)	5.80-7.40	6.17	6.37 ± 0.03	0.04	0.85

a UHM, upper-half mean length of fiber; STR, fiber strength; MIC, micronaire (fiber fineness); UI, fiber uniformity index; ELO, fiber elongation.

### The genetic map and graphical genotypes representing introgression of G. mustelinum in the ILs

**Figure 2:**
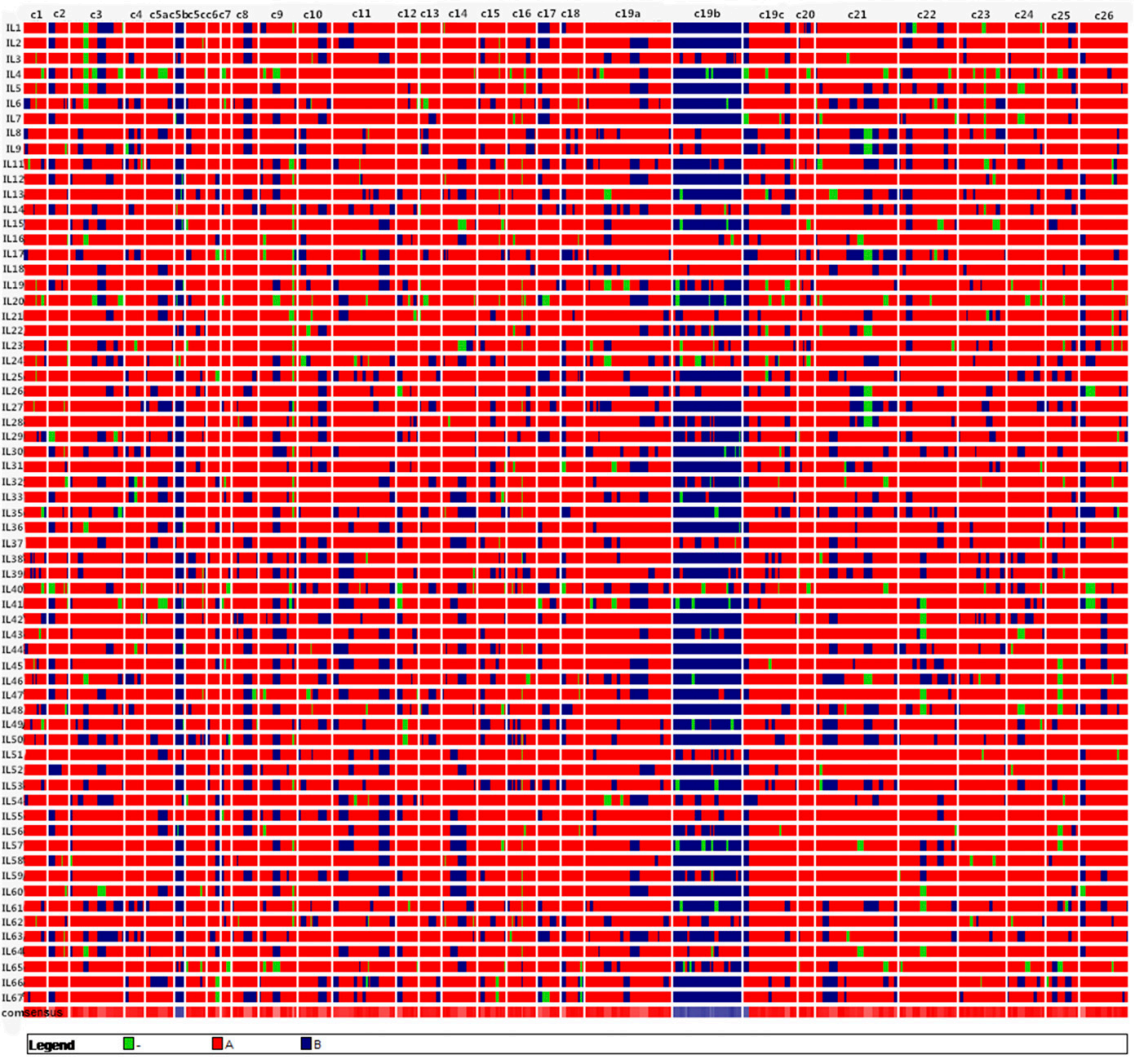
Introgression of *G. mustelinum* in the ILs, monitored as graphical genotypes. (A): *G. hirsutum* homozygote, (B):Homozygous for introgression of *G. mustelinum*, (-): Missing data.

In total, among 1,629 pairs of primers utilized to screen the two parents, PD94042 and *G. mustelinum*, 390 were polymorphic. A genetic linkage map was developed based on the ILs included 346 marker loci and 30 linkage groups, covering a total length of 3,210 cM. The order of markers in this map was congruent with the genetic map of the previously published F_2_ population of *G. mustelinum* by *G. hirsutum* (Wang *et al.* 2016b). Based on this genetic map, graphical genotypes representing the introgression of *G. mustelinum* in the ILs were visualized using GGT2.0. The results showed that *G. mustelinum* introgressions in this set of ILs covered the majority of the cotton genome (Figure 2), with the highest introgression rate on Chromosome 5b and the lowest rate on Chromosome 6, covering 71.6% and 4.4% of the chromosomes, respectively. IL50 and IL16 were identified with the highest and lowest overall amount of *G. mustelinum* chromatin in the IL population, with 23.5% and 5.8% of the genome, respectively.

### QTL detected for fiber quality traits

Composite Interval Mapping detected 49 non-overlapping marker-trait associations in at least one environment for the fiber quality traits (Table 2). The QTL were mapped to 22 chromosomes with 21 located on nine A-genome chromosomes, and 28 on 13 D-genome chromosomes.

**Table 2:** Fiber quality QTL detected by composite interval mapping in *G. mustelinum* ILs

QTL*^a^*	Environment (years)	Flanking markers	Position(cM)	LOD	R^2^ (%)*^b^*	A*^c^*
*qUHM-1-1*	2011	BNL3580-BNL1667	0.01	4.2	10.00	−1.36
	2012	BNL3580-BNL1667	1.01	4.6	39.51	−1.47
qUHM-3-1*^¶¶^	2012	BNL3267-CICR597	44.10	3.0	26.13	−0.85
qUHM-4-1*	2011	BNL530-DPL0667	24.49	8.6	42.66	−2.34
	2012	DPL0085-BNL530	0.01	3.5	20.61	−0.19
qUHM-4-2*	2011	DPL0494-NAU3437	41.46	6.3	18.29	−1.14
qUHM-5-1*^¶^	2011	CICR529-DPL0156	0.01	3.3	9.98	−1.07
*qUHM-10-1*	2012	CICR002a-CICR254	22.90	3.0	23.22	−0.18
*qUHM-11-1^¶¶^*	2011	BNL4094-CICR344	80.22	3.2	9.87	−1.55
*qUHM-13-1*	2011	CICR384-DPL0249b	0.01	4.6	15.74	1.79
*qUHM-16-1*	2012	CICR166b-BNL1604	72.28	3.2	25.23	−0.14
*qUHM-18-1*	2012	BNL1721-MUSS603	37.35	3.1	23.36	−1.42
*qUHM-19-1^¶^*	2012	BNL3811-MUSS118	53.75	3.0	21.62	−1.27
*qUHM-21-1^¶^*	2012	NAU5505-NTU208	213.35	3.0	21.70	−1.38
qUHM-22-1*	2012	NAU3093-CICR345	151.10	3.3	24.84	−0.48
*qUHM-23-1^¶^*	2011	DPL0218-STV022	107.89	3.7	16.28	−1.75
*qUHM-24-1^¶¶^*	2012	CICR673-BNL2772	104.44	3.6	20.58	−4.24
qSTR-11-1*^¶^	2012	DPL0253-NTU150	94.93	7.2	41.78	−5.61
*qSTR-11-2^¶^*	2013	NAU3414-NAU3695	36.31	4.5	21.38	0.55
qSTR-19-1*^¶¶^	2013	MUSS118-CICR782	59.69	4.1	31.35	−3.09
*qSTR-23-1^¶^*	2012	DPL0395-BNL3985	20.48	2.13	29.04	−0.97
	2013	DPL0395-BNL3985	19.48	2.21	31.83	−1.70
*qSTR-25-1^¶^*	2011	NTU191-CICR556	39.38	3.0	22.63	−2.94
qMIC-3-1*	2011	CICR597-DPL0605	51.07	6.5	24.77	0.38
*qMIC-3-2*	2013	NAU2190-BNL3259a	105.23	3.4	42.25	0.59
*qMIC-4-1^¶¶^*	2013	DPL0085-BNL530	3.01	3.1	28.33	0.34
*qMIC-10-1^¶^*	2011	CICR002a-CICR254	26.90	4.5	25.97	0.65
qMIC-11-1*	2012	CICR344-DPL0253	94.76	7.7	32.76	0.93
qMIC-14-1*	2012	NAU862b-NTU182	97.04	6.7	23.03	0.83
*qMIC-15-1^¶¶^*	2013	NTU189-BNL1350	22.00	3.9	32.68	0.53
qMIC-19-1*^¶^	2011	NTU152-BNL3992	81.01	5.5	18.34	0.58
*qMIC-21-1*	2011	CICR559-CICR349	90.35	3.3	12.56	0.15
*qMIC-22-1*	2012	CICR329-NAU2363	110.98	3.4	12.22	−0.41
qUI-3-1*	2011	CICR597-DPL0605	52.07	4.4	22.15	0.84
*qUI-3-2*	2013	NAU2190-BNL3259a	98.23	4.0	23.67	0.93
*qUI-16-1*	2013	CICR166b-BNL1604	67.28	3.8	30.75	1.95
*qUI-17-1*	2013	CICR447-NAU1028	4.01	4.1	25.74	0.17
*qUI-19-1^¶^*	2012	NAU3498-NAU4045b	124.04	3.8	38.48	1.13
*qUI-20-1*	2013	NTU178-BNL3646	30.71	3.5	28.26	2.38
*qUI-22-1*	2013	CICR329-NAU2363	108.98	3.4	29.19	2.86
*qUI-25-1*	201*3*	CICR313-NAU5373	65.64	4.2	26.37	2.65
*qELO-1-1^¶^*	2013	CICR123-CICR105	43.42	3.7	29.27	−0.29
*qELO-9-1^¶¶^*	2013	BNL2590-BNL2750	101.17	4.5	33.83	−0.15
*qELO-10-1*	2013	BNL2960-STV091	96.83	3.4	35.62	0.06
*qELO-12-1*	2013	NAU915-BNL1673	35.95	3.7	24.57	1.33
*qELO-15-1^¶^*	2013	BNL2646-CICR272	60.42	4.2	33.79	−0.67
qELO-18-1*^¶^	2011	CICR316-BNL2652	0.01	3.9	20.95	−0.29
qELO-19-1*^¶^	2011	CICR632-NAU3405b	18.14	5.3	23.21	−0.27
*qELO-19-2^¶^*	2013	BNL3452-CIR165	157.39	4.0	26.03	−0.02
*qELO-21-1^¶^*	2013	NTU165-NAU3341	12.70	3.9	25.73	1.36
*qELO-22-1^¶¶^*	2013	NAU2376-NAU3491	49.44	4.5	30.76	0.02
*qELO-26-1^¶^*	2013	BNL341-NTU214	128.15	4.2	27.33	0.07

a * QTL also detected by single-marker analysis; ^¶^ A QTL was also identified on the same chromosome in our previous studies. ^¶¶^ A QTL was also identified on the same chromosomal region (either associated with the same SSR marker or a nearby marker) in our previous studies.

b R^2^, percentage of phenotypic variation explained by the QTL.

c A, additive effect, a positive number indicates that alleles from the *G. hirsutum* parent increase trait values; a negative number indicates that alleles from the *G. mustelinum* parent increase trait values.

In single-marker analysis, by assuming that each group of consecutive markers showing significant marker-trait association denoted a single QTL, a total of 40 non-overlapping associations were identified, summarized in Table S1. In total, 14 of the 49 significant associations detected by CIM were also detected by single-marker analysis (Table 2) and of the 75 QTL detected by both approaches, the *G. mustelinum* alleles contributed to improved fiber quality for all loci except the QTL for UI associated with the marker CICR597 (Table S1).

### QTL for fiber length

A total of 15 significant associations were detected for fiber length on 14 chromosomes (Table 2), with phenotypic variation explained (PVE) ranging from 9.87% (*qUHM-11-1*) to 42.66% (*qUHM-4-1*). Alleles from PD94042 increased fiber length for one QTL (*qUHM-13-1*), whereas alleles from *G. mustelinum* increased fiber length for the remaining 14 QTL.

### QTL for fiber strength

A total of five significant associations were detected for fiber strength on three chromosomes (Table 2), with PVE ranging from 21.38% (*qSTR-11-2*) to 41.78% (*qSTR-11-1*). Alleles from *G. mustelinum* increased fiber strength for four QTL, namely *qSTR-11-1*, *qSTR-19-1*, *qSTR-23-1*, and *qSTR-25-1*, whereas alleles from Upland cotton increased fiber strength for *qSTR-11-2*.

### QTL for micronaire

A total of 10 QTL were detected on 9 chromosomes for micronaire (Table 2), with PVE ranging from 12.22% (*qMIC-22-1*) to 42.25% (*qMIC-3-2*). Alleles from *G. mustelinum* increased micronaire (undesirable) for *qMIC-22-1*, whereas *G. mustelinum* alleles conferred reduced micronaire (desirable) for the other nine QTL.

### QTL for uniformity index

A total of eight QTL for uniformity index were detected on seven chromosomes (Table 2), with PVE ranging from 22.15% (*qUI-3-1*) to 38.48% (*qUI-19-1*). The favorable alleles of all eight QTL were from the recurrent parent.

### QTL for fiber elongation

A total of 11 QTL for fiber elongation were detected on 10 chromosomes (Table 2), with PVE ranging from 20.95% (*qELO-18-1*) to 35.62% (*qELO-10-1*). Favorable alleles were from *G. mustelinum* for six QTL (*qELO-1-1*, *qELO-9-1*, *qELO-15-1*, *qELO-18-1*, *qELO-19-1*, and *qELO-19-2*), and from the recurrent parent for the remaining 5.

### Stable QTL across years

Of the 49 QTL detected by CIM, two fiber length QTL, *qUHM-1-1* and *qUHM-4-1*, and one fiber strength QTL, *qSTR-23-1* were stably expressed in more than one environment. All the three QTL had high PVE under different environment, with favorable alleles from the *G. mustelinum* parent (Table 2). Composite interval mapping detected the locus *UHM-5-1* in the 2011 dataset and the tightly linked marker CICR529 also showed significant association in single-marker analysis in the same dataset (Table S1). In the 2013 dataset, single-marker analysis did not reach the threshold for declaring a QTL (*P* < 0.001), however it was significant at *P* < 0.005 (data not shown). QTL that are stably expressed across environments with favorable *G. mustelinum* alleles will be of special importance for further research and breeding.

### Consistent QTL validated

QTL detected in this study were compared to our previous QTL analyses in advanced backcross populations (Wang *et al.* 2016a,b; Wang *et al.* 2017a,b). A QTL is validated when it is also identified on the same chromosome regions in our previous studies, either associated with the same SSR marker or nearby markers. Of the 49 QTL detected by CIM, 26 were also detected on the same chromosome in our previous studies, and eight were in the same chromosome regions. These consistent QTL included three for fiber length (*qUHM-3-1*, *qUHM-11-1*, *qUHM-24-1*), two each for micronaire (*qMIC-4-1*, *qMIC-15-1*) and fiber elongation (*qELO-9-1*, *qELO-22-1*), and one for fiber strength (*qSTR-19-1*) (Table 2). The alleles from *G. mustelinum* increased fiber length, strength, and elongation for *qUHM-3-1*, *qUHM-11-1*, *qUHM-24-1*, *qSTR-19-1*, and *qELO-9-1*, respectively, while decreasing micronaire (desirable) and fiber elongation for *qMIC-4-1*, *qMIC-15-1*, and *qELO-22-1*, respectively. Of the eight consistent QTL validated by CIM, two (*qUHM-3-1*, *qSTR-19-1*) were also detected by single-marker analysis. In addition, single-marker analysis found three marker loci associated with fiber QTL in previous studies to also be significantly associated with the same fiber traits in this study (Table S1). The three consistent QTL include the marker locus CICR313 on Chr.25 for micronaire (Wang *et al.* 2017b), DPL0378 on Chr.23 for fiber uniformity (Wang *et al.* 2017a), and CICR204 on Chr.24 for fiber elongation (Wang *et al.* 2016a). Finally, three marker loci associated with fiber QTL in previous studies (BNL2496A on Chr.17 for fiber length, DPL0354 on Chr.03 and BNL3977 on Chr.19 for fiber elongation) did not reach the threshold for declaring a QTL (*P* < 0.001), however they were significant at the *P* < 0.005 level (data not shown).

## Discussion

Upland cotton is faced with a low level of genetic diversity as a result of its evolutionary history, domestication, and modern plant breeding, imposing strong selection pressure on only a small number of agronomic and fiber quality traits (Chandnani *et al.* 2018). A narrow range of phenotypic variation in fiber traits suggests that only a small number of fiber quality genes remain to be recruited for future improvement of the elite cotton gene pool. Several studies have reported on gene introgression from *G. mustelinum* into Upland cotton and the identification of favorable QTL for fiber quality (Wang *et al.* 2016a,b; Shen *et al.* 2017; Wang *et al.* 2017a,b). The current study confirmed prior observations that a small number of ILs carrying different segments of *G. mustelinum* chromatin may possess significantly better fiber quality than both parents. The discovery of such transgressive segregants suggests that introgression of *G. mustelinum* genes has created desirable new gene combinations that positively impact fiber traits in the recurrent *G. hirsutum* parent background. Fiber quality QTL alleles from *G. mustelinum* that improve Upland cotton further support a growing body of evidence that introgressing genes from related species could be a viable approach for improving elite cultivated cotton (Paterson *et al.* 2004).

The two main obstacles in integrating marker-assisted selection to target QTL into the plant breeding toolbox remain the presence of spurious QTL and the inability to accurately determine their position and estimate their effects (Beavis 1994). Therefore, it is critical that the position of a QTL, especially when identified from an early generation segregating population, be validated and its magnitude of effect evaluated in target environments prior to deployment in marker-assisted breeding. This is especially relevant to QTL for traits such as fiber length and strength in interspecific hybrids because such QTL often have unexpected interactions with genetic backgrounds (Chee *et al.* 2005a, 2005b; Draye *et al.* 2005; Brown *et al.* 2019). In addition, genotype × environment interactions have been found for fiber quality traits, reflecting the difficulty in identifying fiber QTL that are stably expressed across environments (Brown *et al.* 2019), as is desirable to cotton breeders for deployment in improved cultivars.

The two most common strategies to validate a QTL position and effect involve generation of additional segregating populations for fine-mapping (Paterson *et al.* 1990) or backcrossing the QTL into one or more genotypes to create near-isogenic lines with and without the favorable allele. Both approaches are time consuming and costly to execute, and in many instances, only able to evaluate one to a few QTL at a time. Nonetheless, using the above strategies, a small number of fiber quality QTL have been validated (Chee and Campbell 2009; Shen *et al.* 2011; Kumar *et al.* 2012; Brown *et al.* 2019). For example, the effect of a fiber length QTL *qFL-chr1*, initially introgressed and mapped in an advanced backcross *G. hirsutum* × *G. barbadense* population (Chee *et al.* 2005b) was confirmed in three independent populations of near-isogenic introgression lines (Shen *et al.* 2011). The position of this QTL was further validated in a large fine-mapping population segregating for the target region derived from a single isogenic line (Xu *et al.* 2017).

However, the total of 131 fiber quality QTL previously identified in the advanced backcross *G. mustelinum* × *G. hirsutum* population, would be laborious and costly to validate individually. In the current study, to validate multiple fiber quality QTL simultaneously in a single experiment, a subset of 65 ILs were selected from an advanced backcross population to carry a unique set of introgression segments but collectively providing wide coverage of the *G. mustelinum* genome. In addition, some ILs were selected based on their fiber quality traits in the BC_3_F_3_ and BC_3_F_4_ (Wang *et al.* 2017a,b); each group of ILs contains lines that showed a significant improvement in at least one fiber quality trait compared to the recurrent parent (Figure 1). Therefore, the population structure for QTL validation herein was similar to those developed for trait-based unidirectional selective genotyping (Lebowitz *et al.* 1987), in which analysis is conducted on a small subset of a large population with high phenotypic values. Simulation studies have shown that selective genotyping of only 10% from a population of 500 lines was adequate to reliably detect a QTL with moderate effect (explains more than 10% phenotypic variance) if a marker was present within 10 cM of the QTL (Lander and Botstein 1989; Navabi *et al.* 2009). Using an empirical QTL mapping dataset from a rice recombinant inbred population of 436 individuals, selective genotyping of only 10 lines each from the upper and lower tails of the phenotypic distribution was sufficient to reliably validate a large-effect QTL conferring drought grain yield (Navabi *et al.* 2009).

Using both CIM and single-marker analysis, a total of 11 QTL, three each for fiber length, micronaire, and elongation and one each for fiber strength and uniformity, were validated (Table 2, Table S1). Three additional putative QTL, one for fiber length and two for elongation, also showed effects significant at the *P* < 0.005 level (Table S1) but failed to meet the stringent *P* < 0.0001 threshold we required. As expected, a majority of the QTL detected have moderate to large effects, with only one (*qUHM-11-1)* explaining less than 10% of the phenotypic variance. While only about 10% (11 at *P* < 0.001 and three at *P* < 0.005) of the 131 QTL previously reported were validated in this study, these results show that selective genotyping has potential for application in which the objective is to validate large numbers of marker-trait associations, especially those with moderate to large-effects, detected for multiple traits simultaneously in one experimental population. However, because of the small sample size and the propensity for segregation distortion, this approach is poorly suited for traits with low heritability or conferred by many small-effect QTL. The main advantage of our approach is that the population size can be quite small because the subset of lines selected to represent the superior or high phenotypic value progenies for one trait (*i.e.*, fiber length) can serve as inferior or low phenotypic value progenies for another trait (*i.e.*, fiber strength). Therefore, for initial QTL validation experiments, only a small number of lines that are most genetically informative in a mapping population are needed for investigation to reduce costs for genotyping and phenotyping. Once a QTL has been validated, a large segregating population can subsequently be developed to fine map the QTL region.

Interestingly, none of the 11 QTL that have been validated showed stable expression in more than one environment. In fact, of the 75 QTL collectively detected by CIM and single-marker analysis, only two QTL for fiber length and one QTL for fiber strength were stably expressed in more than one environment. It should be noted that the genetic background of the ILs, PD19042, was developed from the USDA-ARS Pee Dee breeding program in Florence, South Carolina, and adapted to the Southeastern US cottonbelt. Therefore, the use of ILs with this genetic background, not adapted to the tested environments, could contribute to the observed genotype × environment interactions and exacerbate the lack of stable QTL. It is also plausible that by using only a small number of lines from the extreme tails of a distribution for association analysis, the unbalanced marker allelic frequencies created from selective genotyping can lead to more false associations (Type I error) than the target rate of *P* < 0.001 set in this study (Navabi *et al.* 2009). Therefore, a QTL was declared authentic if a marker locus previously shown to be associated with a fiber QTL also was significantly associated with the same fiber trait in this study. However, other stable marker-trait associations detected in this study would require further validation, so as to exploit some new alleles to improve fiber quality in Upland cotton. Examples of loci falling under this category include *qUHM-1-1*, *qUHM-4-1*, and *qSTR-23-1*, which were detected in two environments, have a high PVE, and with the *G. mustelinum* alleles increased fiber length or strength. Work is now underway to fine-map the regions to validate these loci and to perform transcriptome sequencing to identify candidate genes of fiber quality.

In summary, this study validated exotic QTL for fiber quality identified in our previous study using an advanced backcross population. The selected ILs evaluated herein can be considered permanent genetic resources, unique germplasm for fine mapping and map-based gene cloning to extend our understanding of fiber initiation and development. For cotton breeders who are interested in applying marker-assisted breeding, the genetic markers published previously supplemented by those reported herein would assist in eliminating non-target introgressions by selecting ILs with the least number of introgressions while retaining the QTL regions of interests.
